# Development and Validation of Chinese Health Literacy Scale for Low Salt Consumption - Hong Kong Population (CHLSalt-HK)

**DOI:** 10.1371/journal.pone.0132303

**Published:** 2015-07-06

**Authors:** PH Chau, Angela Y. M. Leung, Holly L. H. Li, Mandy Sea, Ruth Chan, Jean Woo

**Affiliations:** 1 School of Nursing, The University of Hong Kong, Hong Kong, China; 2 Elderly Health Service, Department of Health, Hong Kong, China; 3 Centre for Nutritional Studies, The Chinese University of Hong Kong, Hong Kong, China; 4 Department of Medicine and Therapeutics, The Chinese University of Hong Kong, Hong Kong, China; University Of São Paulo, BRAZIL

## Abstract

Globally, sodium intake far exceeds the level recommended by the World Health Organization. Assessing health literacy related to salt consumption among older adults could guide the development of interventions that target their knowledge gaps, misconceptions, or poor dietary practices. This study aimed to develop and validate the Chinese Health Literacy Scale for Low Salt Consumption - Hong Kong population (CHLSalt-HK). Based on previous studies on salt intake and nutrition label reading in other countries, we developed similar questions that were appropriate for the Chinese population in Hong Kong. The questions covered the following eight broad areas: functional literacy (term recognition and nutrition label reading), knowledge of the salt content of foods, knowledge of the diseases related to high salt intake, knowledge of international standards, myths about salt intake, attitudes toward salt intake, salty food consumption practices, and nutrition label reading practices. Eight professionals, including doctors, nurses, and dietitians, provided feedback on the scale. The psychometric properties of the scale were assessed based on data collected from a convenience sample of 603 Chinese elderly adults recruited from Elderly Health Centres in Hong Kong. The 49-item CHLSalt-HK had a possible score range of 0 to 98, with a higher score indicating higher health literacy related to salt intake. The CHLSalt-HK had acceptable content validity; the item-level Content Validity Index ranged from 0.857 to 1.000, and the scale-level Content Validity Index was 0.994. Additionally, it had good internal consistency (Cronbach's alpha of 0.799) and good test-retest reliability (intraclass correlation coefficient of 0.846). The mean CHLSalt-HK score among those who were aware of the public education slogan about nutrition labels and sodium intake was higher by 3.928 points (95% confidence interval: 1.742 to 6.115) than that among those who were not aware of the slogan, which supports adequate discriminant validity. The validated CHLSalt-HK had acceptable content validity, acceptable construct validity, good internal consistency, good test-retest reliability, and adequate discriminant validity. The scale could be completed in 10-15 minutes and is easy to administer compared with the collection of biomarkers or food diaries. Further research should investigate its concurrent validity and predictive validity. The development of this scale supports the first step in salt intake reduction among older Chinese adults in Hong Kong by enabling the assessment of their health literacy related to salt consumption in health screenings or health assessments, and it can be used to evaluate salt reduction interventions.

## Introduction

Hypertension is a common chronic illness among older people. There is an essential need for population control of hypertension because it is a major contributor to cardiovascular disease. One of the effective ways to control hypertension is by reducing salt intake.[1] However, the daily salt intake worldwide, particularly in Asian countries, was approximately 9 g to 12 g, which is more than doubled the World Health Organization (WHO) recommendation.[2] Therefore, a population reduction in salt intake is of high priority. Reduction through individual and food industry interventions is recommended for the population control of hypertension.[1,3] However, before developing interventions that promote behavioral change related to dietary salt intake, it would be useful to assess health literacy related to salt consumption among older adults, so that the interventions can be more targeted in terms of knowledge gaps, misconceptions, or poor dietary practices.

According to the United States Centers for Disease Control and Prevention (CDC), health literacy is defined as “the degree to which an individual has the capacity to obtain, communicate, process, and understand basic health information and services to make appropriate health decisions”.[4] To date, there is no standard definition of health literacy, but a recent review reported that the following three domains were included in all of the definitions and concepts that were reviewed: functional literacy, factual and procedural knowledge, and awareness.[5] Adults who have low health literacy have difficulty understanding, processing and implementing the healthcare information that they receive, and they may ultimately fail to effectively manage their health. Another recent review showed that low health literacy was associated with poorer health outcomes and increased healthcare utilization.[6] Furthermore, older adults are at higher risk of low health literacy compared with individuals in other age groups.[7]

In the context of health literacy related to low salt consumption, the basic health information includes the salt content of different types of food and the adverse effects of high salt consumption on health. The lay public may not even know the difference between sodium and salt.[8] Though sodium content is printed on the nutrition labels of prepackaged foods, individuals often have difficulties understanding the information available on the nutrition labels.[9] For foods that do not have nutrition labels, individuals must rely on the information obtained through public education, mass media, peer communications, or their own experiences to determine whether the salt content is high. However, the lay public was found to often perceive their knowledge level about dietary salt intake to be low to medium.[10] Furthermore, although the majority of people could associate high salt intake with an increased risk of kidney disease, only a small proportion were aware of the association between high salt intake and increased risk of heart disease or bone disease.[11] This often results in inappropriate decisions regarding the dietary intake of salt.

In Hong Kong, a city that has a rapidly aging Chinese population, the prevalence of hypertension among men and women aged 65–74 years increased from 52% and 55%, respectively, in 1995–96 to 66% and 70%, respectively, in 2003–04.[12,13] It has been shown that the trends in stroke incidence coincided with the increasing trends in hypertension.[14] Meanwhile, the sodium intake of the local population far exceeds the level recommended by the WHO. In 2011, approximately 82% of postmenopausal women consumed more than 5 g of salt per day.[15] This proportion is quite similar to that found in an earlier study in 1995–96, which found that 78% of men and women in Hong Kong had a daily sodium intake of more than 2300 mg.[16] There is a lack of not only surveys about knowledge, attitudes and dietary practices related to low salt consumption in Hong Kong but also a validated scale on health literacy related to low salt consumption. On the contrary, studies have examined the knowledge, attitudes and practices related to salt consumption in the United States and Australia.[8,10,11] However, the results may not be generalizable to the Hong Kong population because dietary habits differ between Eastern and Western societies. Therefore, this study aimed to develop and validate the Chinese Health Literacy Scale for Low Salt Consumption—Hong Kong population (CHLSalt-HK) to facilitate the development of future interventions on the important public health issue of high salt consumption.

## Materials and Methods

### Target population

The target population was older Chinese adults. The inclusion criteria were as follows: i) being at least 65 years of age; ii) being a Chinese Hong Kong resident (i.e., living in Hong Kong for at least three months each year); and iii) having the ability to speak and understand Cantonese. The exclusion criteria were as follows: i) having cognitive impairment and ii) having any communication problems due to functional impairment (e.g., blindness, deafness, aphasia). The Short Portable Mental Screening Questionnaire (SPMSQ) [17,18] was used to screen for cognitive impairment, and individuals who scored 7 or below were excluded.

A sample size of 540 subjects was required to support a ratio of 10 subjects per item on the scale to be validated [19]. Furthermore, assuming that 10% of the item-wise values would be missing, a sample of 600 subjects was needed. Among these subjects, 41 participants were interviewed again after two weeks to estimate test-retest reliability, and 40 participants responded to a self-administered questionnaire before they were interviewed (by a trained research assistant) to estimate inter-rater reliability.

### Data collection

A cross-sectional survey was conducted in four randomly selected Elderly Health Centres (EHCs) in Hong Kong. The Department of Health has established 18 EHCs in Hong Kong to promote the health of Hong Kong residents aged 65 years and older through a multi-disciplinary team approach in a primary care setting. The services provided by the EHCs include health assessments, physical check-ups, individualized health counseling, health education and general outpatient services. EHC members receive a comprehensive health assessment upon their enrollment or renewal of membership. Members can also visit the EHCs for primary care services (e.g., annual influenza vaccinations, blood pressure monitoring) and secondary care services (e.g., medical consultations for influenza symptoms). We used a cluster sampling method to randomly select four centers out of the 18 EHCs, one from each region of Hong Kong (i.e., Hong Kong Island, Kowloon, New Territories East, and New Territories West). Because one of the randomly selected EHCs was too small to accommodate the research team, another center was randomly selected from the same region. Members who visited the selected EHCs from February to August 2014 were invited to participate in the study.

At the EHCs, convenience sampling was employed. After the EHC members were seen by the doctors and/or the nurses, the research assistants explained the objectives and procedures of the study, screened members for eligibility, and sought informed consent for the members’ participation in the study. A travel card holder was given to each participant as a token of appreciation for their participation.

### The instrument

A structured questionnaire was used during the interviews.

First, the CHLSalt-HK was validated. The draft version of the CHLSalt-HK was developed according to the three most common domains of health literacy identified by Frisch et al.: functional literacy, factual and procedural knowledge, and awareness.[5] Based on previous studies on knowledge, attitudes and dietary practices related to salt consumption and nutrition label reading in other countries,[8,10,11,20] we developed similar questions that were appropriate for the Chinese population in Hong Kong. In addition, some myths about daily dietary salt intake [21] were covered as part of the knowledge domain. The questions covered the following eight broad areas: functional literacy (term recognition and nutrition label reading), knowledge of the salt content of foods, knowledge of the diseases related to high salt intake, knowledge of international standards, myths about salt intake, attitudes toward salt intake, salty food consumption practices, and nutrition label reading practices. The responses to the questions were in the forms of either a 5-point Likert scale or 4 multiple-choice options. For the Likert scale questions, a score of 2 was assigned to the most favorable option, a score of 1 was assigned to the next favorable option, and a score of 0 was assigned to the remaining three options. For the multiple-choice questions, a score of 2 was assigned to the correct answer, and a score of 0 was assigned to the remaining options. The total score was calculated by summing up the scores on each item. The draft version of the scale was reviewed by an expert panel of eight professionals, including doctors, nurses, and dietitians. To guide appropriate modification of the scale, each expert rated the relevance of each item on a 4-point Likert scale (1 = very irrelevant; 4 = very relevant). The item-level Content Validity Index (CVI) was calculated as the proportion of 3 or above ratings by the panel members, and an item-level CVI of 0.78 or above was considered as acceptable.[22] The panel also suggested additional items if they were not covered. An adjusted draft version with 54 items was developed and piloted with a sample of 17 elderly adults. Based on this pilot sample, the wording was fine-tuned to increase the readability of the questions and to minimize misinterpretations. The validation of the scale is based on this fine-tuned version.

Second, a validated Chinese Health Literacy Scale for Chronic Care (CHLCC) was included in the structured questionnaire to assess the convergent validity of the CHLSalt-HK. The CHLCC was developed to evaluate the health literacy level of patients with chronic illness, including diabetes, hypertension, chronic obstructive airway disease, and arthritis, and it has good psychometric properties.[23] The CHLCC assesses the following four skills related to chronic care: 1) remembering (i.e., the ability to read aloud terms that are often used in chronic care); 2) understanding (i.e., the ability to understand instructional messages); 3) applying (i.e., executing procedures in a given situation); and 4) analyzing (i.e., breaking materials into parts and determining how the parts are inter-related). Because both scales aim to measure health literacy among the Chinese population, they were expected to be correlated to some extent. However, though both the CHLCC and the CHLSalt-HK are health literacy scales, their focuses are slightly different; the CHLCC focuses more on medical instruction and medication-taking practices, and the CHLSalt-HK focuses more on daily life experiences. Therefore, the correlation should be low.

Third, data on demographics and medical history were collected from the subjects. The variables included age, sex, education level, and presence of chronic illnesses. In addition, the subjects were also asked whether they had heard of the public health slogan “Compare Nutrition Labels Side by Side to Choose the Lower Sodium Options,” which was used by the Centre for Food Safety in Hong Kong to inform the public about comparing nutrition labels to reduce salt intake.

### Statistical analysis

Descriptive statistics of the respondents’ characteristics were tabulated. The mean and standard deviation (SD) of each respondent’s CHLSalt-HK score were calculated, and the relationships between the scores and the demographic variables were assessed with either a t-test or a one-way ANOVA, depending on which was appropriate.

The scale’s psychometric properties, including construct validity, content validity, internal consistency, test-retest reliability, inter-rater reliability, convergent validity, discriminant validity, and floor/ceiling effects, were assessed. The construct validity was assessed by a second-order confirmatory factor analysis with categorical indicators. The model adequacy was assessed by the two-index combination strategy that was proposed by Hu and Bentler.[24] A model with a root mean square error of approximation (RMSEA) ≤ 0.06 and a standardized root mean square residuals (SRMR) ≤ 0.09 was considered adequate. A comparative fit index (CFI) was also calculated; values above 0.90 indicated an acceptable fit, and values above 0.95 indicated a good fit.[24] The content validity was assessed by an item-level CVI and a scale-level CVI. The item-level CVI was calculated as the proportion of 3 or above ratings by the panel members, and a value of 0.78 or above was considered acceptable.[22] The scale-level CVI was the average of the item-level CVI, and a value of 0.9 or above was considered to be acceptable.[22] The internal consistency was assessed by Cronbach’s alpha, and a value above 0.7 was considered to be acceptable.[19] The test-retest reliability was assessed by the Intraclass Correlation Coefficient (ICC) between the responses that were collected two weeks apart. The inter-rater reliability was assessed by the ICC between the responses collected through two different modes of questionnaire administration (i.e., self-administered vs. face-to-face interview). A two-way mixed effects, absolute agreement, single measure ICC was used. A value above 0.7 was considered to be acceptable.[19] The convergent validity was assessed by correlation analysis between the CHLSalt-HK and CHLCC scores. The discriminant validity was assessed by using the two-independent samples t-test to evaluate the following differences: 1) differences in the CHLSalt-HK scores between those with hypertension and those without hypertension and 2) differences in the CHLSalt-HK scores between those who were aware of the public education slogan and those who were not aware of the slogan. The standardized effect sizes of the differences were also calculated based on a Cohen’s d. A value of 0.2 indicated a small effect, a value of 0.5 indicated a medium effect and a value of 0.8 indicated a large effect.[25] The floor or ceiling effects were assessed by the proportion of respondents who obtained the lowest and the highest scores. All analyses were conducted using SPSS version 20 (IBM Corp, Armonk, NY), with the exception of the confirmatory factor analysis, which was conducted using MPlus version 7 (Muthén & Muthén, Los Angeles, CA):.

### Ethics statement

This study received ethics approval from (i) the Institutional Review Board of the University of Hong Kong/Hospital Authority Hong Kong West Cluster, and (ii) the Ethics Committee of Department of Health of The Government of Hong Kong Special Administrative Region. Written informed consent was obtained from participant.

## Results

**Fig 1:**
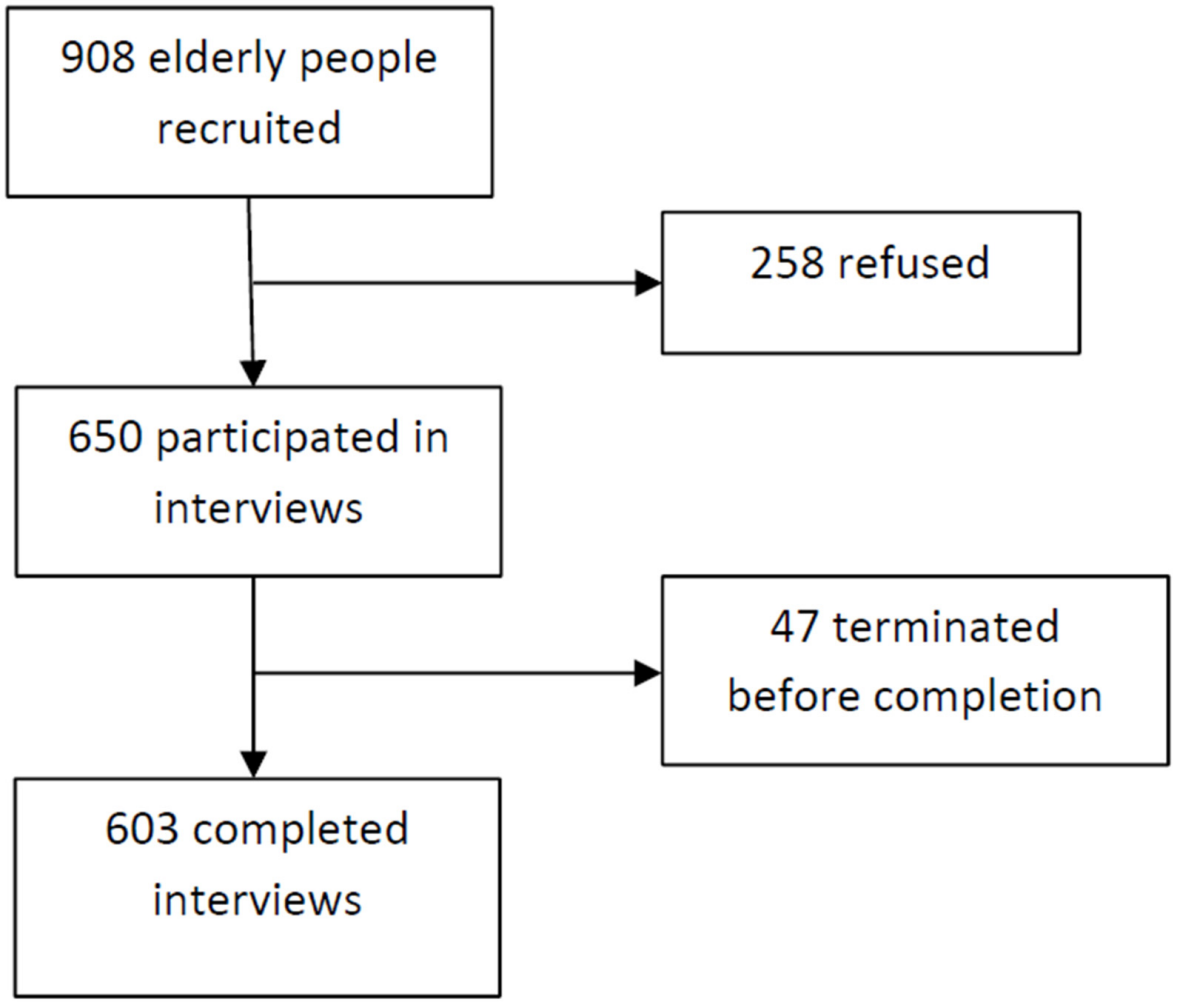
Recruitment of participants in the study

A total of 908 elderly adults were recruited at the EHCs. Of those recruited, 258 refused to participate, and 47 did not complete the interview (i.e., the respondents left the EHC before the end of the interview due to other engagements). In total, 603 interviews were completed, with a response rate of 66.4%. Fig 1 shows the recruitment of participants in the study. Data at the item level were missing in only 1.3% of the 603 valid interviews. Table 1 shows the respondents’ characteristics. The respondents had a mean age of 76.5 years (SD = 5.8), and 55.9% were females. Approximately 45.3% of the respondents had no formal education or an education level between 1^st^ and 6^th^ grade, 38.3% had an education level between 7^th^ and 12^th^ grade, and 16.4% had an education level above 12^th^ grade. Over half of the respondents (52.9%) reported being previously diagnosed with hypertension by a doctor, and approximately 52.1% were the main person who cooked for the family.

**Table 1:** Characteristics of the respondents (n = 603).

Characteristics	n (percent)
Age (years), mean ±SD	76.5±5.8
Sex	
Male	266 (44.1)
Female	337 (55.9)
Educational Level	
No formal education or between 1^st^ and 6^th^ grade	273 (45.3)
Between 7^th^ and 12^th^ grade	231 (38.3)
Above 12^th^ grade	99 (16.4)
Occupation	
Retired	482 (79.9)
Home-maker/Housewife	110 (18.2)
Others	11 (1.8)
Monthly household income	
< HKD15,000	434 (72.0)
≥ HKD15,000	153 (25.4)
Missing	16 (2.7)
Hypertension	
Yes	319 (52.9)
No	278 (46.1)
Missing	6 (1.0)
Heart diseases	
Yes	64 (10.6)
No	538 (88.9)
Missing	3 (0.5)
Stroke	
Yes	25 (4.1)
No	577 (95.7)
Missing	1 (0.2)
Diabetes	
Yes	95 (15.8)
No	504 (83.6)
Missing	4 (0.7)
Renal diseases	
Yes	10 (1.7)
No	591 (98.0)
Missing	2 (0.3)
Osteoporosis	
Yes	159 (26.4)
No	440 (73.0)
Missing	4 (0.7)
Main person who cook for the family	
Yes	314 (52.1)
No	289 (47.9)
Main person who purchase cooking ingredients for the family	
Yes	343 (56.9)
No	260 (43.1)

Note: Participants who completed the CHLSalt-HK but had missing data on the demographics variables were included in the analysis if the missing value was not involved in the respective analysis.

### Construct validity

**Fig 2:**
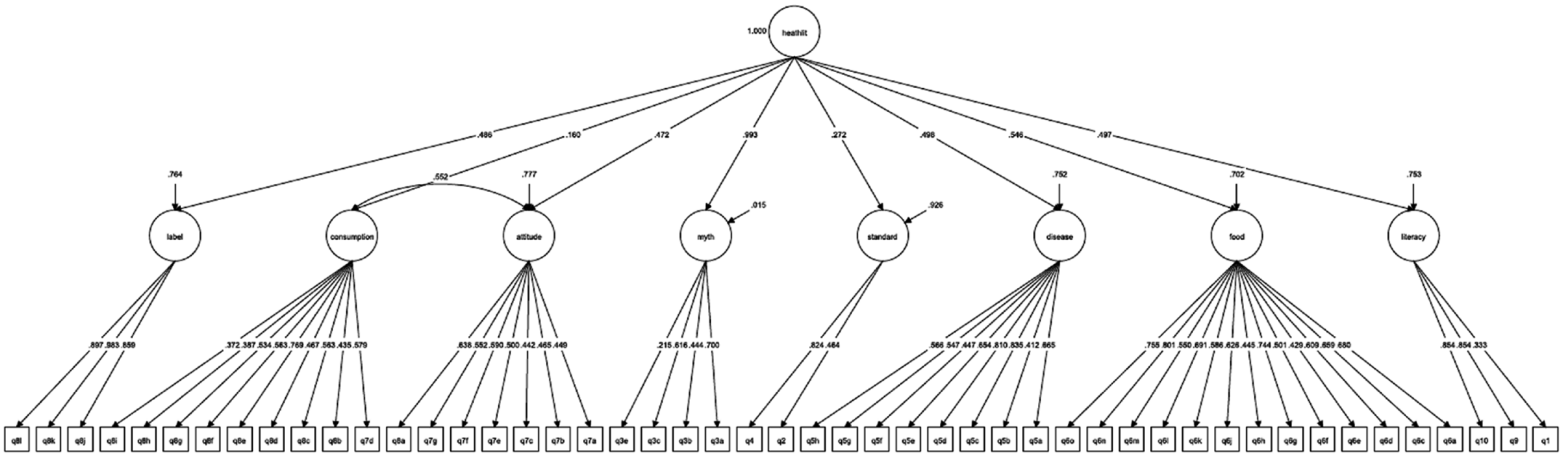
Factor structure of the Chinese Health Literacy Scale for Low Salt Consumption—Hong Kong population (CHLSalt-HK).

A factor structure with 54 items in eight first-order factors and one second-order factor was examined. However, the structure did not show adequate fit (RMSEA = 0.033, 90% confidence interval [CI]: 0.031 to 0.036; SRMR = 0.088; CFI = 0.871). Thus, the items with insignificant loading or with a loading of less than 0.2 in the first-order factors were removed from the model, leaving 49 items. The eight first-order factors were functional literacy (3 items), salty food knowledge (13 items), disease knowledge (8 items), knowledge of international standards (2 items), myths about salt intake (4 items), salt intake attitudes (7 items), salty food consumption (9 items), and nutrition label practices (3 items). Table 2 shows the 49 items in the eight first-order factors. The second-order factor was health literacy related to low salt intake. Model modification was driven by the modification index, which suggested that there was a correlation between the salt intake attitudes and the salty food consumption factors. The final model had a RMSEA of 0.033 (90% CI: 0.030 to 0.035) and a SRMR of 0.085, which indicates adequate fit.[24] The CFI was 0.901, which also indicates acceptable fit. This structure confirmed that all 49 of the items were measuring the same health literacy domain. Fig 2 shows the factor structure and the standardized estimates of the model.

**Table 2:** Questions and factors on the Chinese Health Literacy Scale for Low Salt Consumption—Hong Kong version (CHLSalt-HK) and standardized loadings.

Factor/Question
Functional literacy (3 items)
Q1 Which of the following statements best describes the relationship between salt and sodium?
Q9 Refer to the following nutrition labels of various biscuits. Which type of biscuits would you choose if you wish to minimize salt intake? [shown with the most basic nutrition labels]
Q10 Refer to the following nutrition labels of various canned soups. Which of the canned soups has the highest salt content? [shown with nutrition labels with additional information]
Salty food knowledge (13 items)
Q6 Please indicate whether the salt content of the foods listed below is low, medium or high. Please refer to the same amount of food (for example, 100 g) and choose one answer for each food.
a. Lunch meat
b. Guangdong BBQ Pork (with sauce)
c. Potato chips
d. White sliced bread
e. Corn flakes
f. Instant noodles (with seasoning powder)
g. Sliced cheese
h. Pork Siu Mai (Chinese pork dim sum)
i. Ketchup
j. Oyster sauce
k. Salad dressing
l. Hamburger
m. Pizza
Disease knowledge (8 items)
Q5 Do you agree that the following illnesses can be caused by high salt intake?
a. High blood pressure
b. High blood sugar
c. Heart diseases
d. Stroke
e. Kidney disease
f. Osteoporosis
g. Stomach cancer
h. Obesity
Knowledge of international standards (2 items)
Q2 How many grams of salt does one teaspoon of salt have?
Q4 What is the daily limit of salt intake (in grams) that is recommended by the World Health Organization for an adult?
Myths about salt intake (4 items)
Q3 Please indicate how you think about the following statements:
a. Only by adding salt and sauces while cooking can the taste of food be enhanced
b. Sodium intake can be reduced by replacing salt with plenty of chicken powder during cooking
c. Most foods available at restaurants (e.g., Chinese restaurants, fast food restaurants) are high in salt
d. Drinking more water can neutralize salt intake from my diet
Salt intake attitudes (7 items)
Q7 Please indicate how you think about the following statements:
a. I worry about the serious health problems that are caused by eating salty foods
b. Most low salt foods taste bad
c. I feel too much pressure to eat a healthy diet
e. Limiting the amount of salt intake is important to my health
f. I am concerned about the salt content in foods
g. I am confident that I can control my daily salt intake
Q8a Please indicate how often do you minimize salt intake.
Salty food consumption (9 items)
Q7d Please indicate how you think about the statement “I enjoy eating salty foods.”
Q8 Please indicate how often you do the following:
b. Add salt at the table
c. Add sauce or condiments (e.g., soya sauce, liquid seasoning, oyster sauce, chili sauce) at the table
d. Consume canned foods
e. Consume salted fish, salted vegetables, salted duck eggs or preserved meats
f. Consume Guangdong BBQ meat
g. Consume salted snacks (e.g., beef jerky, salted nuts, roasted squid floss)
h. Consume preserved fruits (e.g., preserved orange peels, preserved plums, raisins)
i. Consume fast food
Nutrition label practices (3 items)
Q8 Please indicate how often you do the following:
i. Pay attention to whether the food is labeled as “No added salt” or “Low in salt”
j. Read the sodium content stated on the nutrition labels on food packages
k. Purchase foods according to the sodium content stated on the nutrition labels

### Descriptive statistics of CHLSalt-HK

The final version of the CHLSalt-HK consisted of 49 items and had a possible score range of 0 to 98. A higher score indicated higher health literacy related to low salt intake. Our sample of elderly Chinese adults had a mean score of 60.042 and a standard deviation of 10.794. The median score was 61, the observed minimum score was 8 and the observed maximum score was 87. None of the respondents scored the maximum of 98 or the minimum of 0; therefore, floor or ceiling effects likely did not occur.

Table 3 shows the mean CHLSalt-HK score and its relationships with the demographic variables. The CHLSalt-HK score decreased with age (Pearson correlation coefficient of -0.221, p<0.001). Those with no formal education or an education level between 1^st^ and 6^th^ grade had a significantly lower CHLSalt-HK score than those with an education level between 7^th^ and 12^th^ grade by 3.760 (95% CI: 1.480 to 6.039) and those with an education level above 12^th^ grade by 5.953 points (95% CI: 2.950 to 8.955). Women had a significantly higher CHLSalt-HK score than men by 1.863 points (95% CI: 0.117 o 3.609). There was no significant difference in CHLSalt-HK score between those shared the majority of the responsibility for cooking for the family and those who did not (p = 0.489).

**Table 3:** Mean and standard deviation (SD) CHLSalt-HK score and its relationships with demographic variables.

Characteristics	Mean (SD)	p-value^a^
Total (n = 595)	60.042 (10.794)	
Age group		<0.001
65–74 (n = 218)	63.106 (10.306)	
75–84 (n = 328)	58.485 (10.578)	
≥85 (n = 49)	56.837 (11.384)	
Sex		0.037
Male (n = 261)	58.996 (11.116)	
Female (n = 334)	60.859 (10.480)	
Educational Level		<0.001
No formal education or between 1^st^ and 6^th^ grade (n = 269)	57.625 (10.425)	
Between 7^th^ and 12^th^ grade (n = 229)	61.384 (10.630)	
Above 12^th^ grade (n = 97)	63.577 (10.760)	
Hypertension		0.037
Yes (n = 315)	59.254 (10.990)	
No (n = 276)	61.098 (10.376)	
Main person who cook for the family		0.489
Yes (n = 309)	60.337 (10.405)	
No (n = 286)	59.724 (11.208)	

Note: Due to missing item-level data, the sample size was less than 603. The sample size in each group is given in parentheses.

^a^. Based on t-test or one-way ANOVA

### Content validity

The final version of the CHLSalt-HK had an item-level CVI that ranged from 0.857 to 1.000 and a scale-level CVI of 0.994. Both the item-level and the scale-level CVI indicated adequate content validity.[22]

### Internal consistency

The 49-item CHLSalt-HK had a Cronbach's alpha of 0.799, which indicates good internal consistency.[19] However, the internal consistency of the eight factors ranged from 0.394 to 0.855; therefore, the use of subscales cannot be considered for this scale.

### Test-retest reliability

A total of 41 respondents were interviewed twice, at least two weeks apart. The ICC was found to be 0.846 (95% CI: 0.707 to 0.919), which indicates good test-retest reliability.

### Inter-rater reliability

A total of 40 respondents completed both a self-administered questionnaire and a face-to-face interview. Due to item-level data missing, only data from 38 respondents could be used to assess inter-rater reliability. The ICC was found to be 0.700 (95% CI: 0.457 to 0.839). Due to the wide confidence interval, the inter-rater reliability assessment was inconclusive.

### Convergent validity

The CHLSalt-HK was significantly correlated with the CHLCC. The Pearson correlation coefficient between the two scales was 0.288 (p < .001). Convergent validity was not supported.

### Discriminant validity

The mean (SD) CHLSalt-HK score was 61.098 (10.376) among those without hypertension and 59.254 (10.990) among those with hypertension. Those without hypertension had a significantly higher CHLSalt-HK score than those with hypertension by 1.844 points (95% CI: 0.110 to 3.578). This difference was equivalent to a very small effect size (Cohen’s d = 0.171), which indicates that there is a likely association between hypertension and lower health literacy related to salt intake. The mean CHLSalt-HK score also differed between those who had heard of and those who had not heard of the Chinese public health slogan “Compare Nutrition Labels Side by Side to Choose the Lower Sodium Options” from the Centre for Food Safety in Hong Kong. The mean (SD) CHLSalt-HK score was 63.140 (9.616) among those who had heard of the slogan and 59.212 (10.907) among those who had not heard of the slogan. Those who had heard of the slogan scored significantly higher by 3.928 points (95% CI: 1.742 to 6.115) than those who had not heard of the slogan. This was equivalent to a small-to-medium effect size (Cohen’s d = 0.364), which suggests that there is an association between higher health literacy related to salt intake and more awareness of public health education related to low salt intake.

## Discussion

The CHLSalt-HK is the first validated scale for assessing health literacy related to low salt intake among Chinese elderly adults in Hong Kong. The 49-item scale has a possible score range from 0 to 98, and a higher score indicates higher health literacy related to low salt intake. The results of this study indicate that the validated CHLSalt-HK has acceptable psychometric properties. It is more advantageous than using biomarkers or food diaries because it is easy to administer and only takes 10 to 15 minutes to complete.

Based on the construct validity analysis, the item “I enjoy eating salty foods” was included in the “salty food consumption” factor. In contrast, the item “how often do you minimize salt intake” was included in the “salt intake attitudes” factor. However, combining the two factors or switching the two items did not improve the model fit. Instead, the modification index suggested the two factors “salty food consumption” and “salt intake attitudes” are correlated. This finding indicates that salt intake consumption and attitudes might not be distinct and may reflect the inter-relationship between attitudes and practices. Although the CHLSalt-HK had an eight-factor structure, the Cronbach’s alpha was below the commonly accepted threshold of 0.7 for some of the factors.[19] There are many possible reasons for a low alpha value, such as too few questions, poor inter-relation between items and heterogeneous constructs.[26] We could not conclude that the factors had poor internal consistency; however, without evidence of acceptable internal consistency, we recommend that the total scale score be used instead of the subscale scores.

To explore the validity of the scale when it was self-administered, the ICC was calculated between the scores obtained when it was administered through a face-to-face interview and the scores that were obtained when it was self-administered. Although the point estimate just met the acceptable level of 0.7, the confidence interval was too wide to draw any conclusions about the validity. Further research is needed to reassess this property with a larger sample. There were two respondents with item-level data missing on the self-administered questionnaire. If the missing item was replaced by a score of zero, the ICC was even lower, which suggests that the missing values should not be included.

Although discriminant validity was established between those who had heard of the public education slogan and those had not heard of the slogan (with a small-to-medium effect size), the difference between the hypertensive and non-hypertensive groups was smaller than expected (with less than a small effect size). One explanation may be that the respondents who were diagnosed with hypertension received health education about disease management that included the lowering of salt intake, and this might have raised their health literacy. The next step should be to investigate the predictive validity of the scale. A follow-up study has been planned to examine the hypertension status of the respondents who did not have hypertension at the time of this study in one or two years. Moreover, a cut-off score on the CHLSalt-HK will be established to distinguish individuals who are at high risk for developing hypertension.

The convergent validity of the scale was assessed by the correlation between the CHLSalt-HK scores and the CHLCC scores. Although both the CHLSalt-HK and the CHLCC are health literacy scales, their focuses are slightly different. As expected, there was a low but significant correlation between the two scales, and thus, the convergent validity results are not very promising. To date, measuring 24-hour urinary sodium excretion is regarded as the gold standard for assessing dietary salt intake [27]. It would be worthwhile to establish the concurrent validity of the CHLSalt-HK with this measure. Given that concurrent validity has been established, the CHLSalt-HK could be a non-biological measure of dietary salt intake that is used as a tool to evaluate the effectiveness of salt intake reduction interventions.

From a public health literacy perspective,[28] excessive salt intake is no longer an individual preference or choice but a decision that is made by individuals or groups on the behaviors of individuals for the benefit of the community. Consuming too much salt in our daily diet increases the chance of developing hypertension, which has been shown to eventually cause cardiac diseases, a relationship that is well-established.[29] Moreover, evidence shows that the adverse effects of high salt intake not only increases the risks of cardiovascular diseases but also a wide variety of other diseases such as renal diseases, obesity, osteoporosis, and stomach cancer.[1] To support individuals in making the appropriate decisions related to dietary salt consumption, the initial step may be to build an individual’s capacity to receive and interpret the health information on nutrition labels or educational brochures with dietary advice; that is, to improve their health literacy related to salt intake. Subsequently, community leaders could increase public awareness about salt intake and develop public discussions about the ways to reduce salt in food production, particularly in the food and catering industries. By now, reduced salt diets have been advocated for in different countries; however, their actual implementation has been rare.[30] The development of the CHLSalt-HK supports the first step in salt intake reduction among older Chinese adults in Hong Kong by enabling the assessment of their health literacy related to salt consumption in health screenings or health assessments, and it can be used to evaluate salt reduction interventions.

The CHLSalt-HK was developed in a sample of elderly Chinese adults in Hong Kong. Among the 49 items, 29 items were not related to specific foods. Among the remaining 20 items that mentioned specific foods, 13 items included foods that are globally available (e.g., sliced cheese, pizza, hamburgers) and only 7 items included Chinese foods (e.g., Guangdong BBQ pork, siu mai [a type of dim sum], oyster sauce, preserved meat/vegetables, Chinese style dried food). These culture specific items could be replaced with the salty foods that are common in different countries. Two nutrition labels were included in this scale to test the subjects’ nutrition label reading skills. To use this scale in another city, the nutrition labels should be adapted to be the same format as what is most commonly used or what is required by law in the local context. For non-Chinese speaking communities, the scale can be translated to different languages with local adaptations. Interested parties should contact the corresponding author for the details of the questionnaire.

While elderly adults are the priority target group of salt reduction interventions, the health literacy of other age groups should also be addressed in future studies. In a sample of 72 Chinese subjects aged 18 to 64 years from another study (not published), it was found that the CHLSalt-HK has a Cronbach's alpha of 0.791, which indicates satisfactory internal consistency among individuals who are younger than 65 years of age. In the current study, the CHLSalt-HK was validated among elderly adults; thus, future studies may focus on validating the scale in younger age groups.

This study has several strengths. First, it involved a panel of eight experts in developing the CHLSalt-HK. Second, a large sample of 600 subjects was recruited to validate the scale. Third, a wide range of psychometric properties was assessed. However, there are limitations related to the study design. The results may be subject to selection bias because convenience sampling was used. EHC members might have higher health literacy than the general elderly population because they have access to primary health care. Additionally, the self-reported nature of the responses may be subject to response bias if the subjects did not wish to disclose their unhealthy dietary practices, such as the frequent consumption of salty foods. The Classical Test Theory was used to develop the scale; stronger approaches such as the Grounded Theory or the Item Response Theory should be considered in future studies. While ample questions on knowledge and awareness were included in the scale, only three items focused on functional literacy; thus, the functional literacy domain might be underrepresented in the scale.

## References

[1] He FJ, MacGregor GA. Reducing population salt intake worldwide: from evidence to implementation. Prog Cardiovasc Dis 2010; 52: 363–382. 10.1016/j.pcad.2009.12.006 20226955

[2] Brown IJ, Tzoulaki I, Candeias V, Elliott P. Salt intakes around the world: implications for public health. Int J Epidemiol 2009; 38: 791–813. 10.1093/ije/dyp139 19351697

[3] Webster J, Trieu K, Dunford E, Hawkes C. Target salt 2025: a global overview of national programs to encourage the food industry to reduce salt in foods. Nutrients 2014; 6: 3274–3287. 10.3390/nu6083274 25195640 PMC4145308

[4] Centers for Disease Control and Prevention. Health Literacy. 2014; Available: http://www.cdc.gov/healthliteracy/ Accessed on 3 Dec 2014.

[5] Frisch AL, Camerini L, Diviani N, Schulz PJ. Defining and measuring health literacy: how can we profit from other literacy domains? Health Promot Int 2012; 27: 117–126. 10.1093/heapro/dar043 21724626

[6] Berkman ND, Sheridan SL, Donahue KE, Halpern DJ, Crotty K. Low health literacy and health outcomes: an updated systematic review. Ann Intern Med 2011; 155: 97–107. 10.7326/0003-4819-155-2-201107190-00005 21768583

[7] Cutilli CC, Schaefer CT. Case studies in geriatric health literacy. Orthop Nurs 2011; 30: 281–285; quiz 286–287. 10.1097/NOR.0b013e3182247c8f 21799388

[8] Claro RM, Linders H, Ricardo CZ, Legetic B, Campbell NR. Consumer attitudes, knowledge, and behavior related to salt consumption in sentinel countries of the Americas. Pan American Journal of Public Health 2012; 32: 265–273. 23299287 10.1590/s1020-49892012001000004

[9] Temple NJ, Fraser J. Food labels: a critical assessment. Nutrition 2014; 30: 257–260. 10.1016/j.nut.2013.06.012 24139165

[10] Sarmugam R, Worsley A, Flood V. Development and validation of a salt knowledge questionnaire. Public Health Nutr 2014; 17: 1061–1068. 10.1017/S1368980013000517 23507427 PMC10282258

[11] Kim MK, Lopetcharat K, Gerard PD, Drake MA. Consumer awareness of salt and sodium reduction and sodium labeling. J Food Sci 2012; 77: S307–313. 10.1111/j.1750-3841.2012.02843.x 22957915

[12] Department of Health of HKSAR, Department of Community Medicine of The University of Hong Kong. Report on Population Health Survey 2003/2004. 2005; Hong Kong: HKSAR and The University of Hong Kong.

[13] Janus ED. The Hong Kong Cardiovascular Risk Factor Prevalence Study 1995–1996. 1997; Hong Kong: Cardiovascular Risk Factor Prevalence Study Group.

[14] Chau PH, Woo J, Goggins WB, Tse YK, Chan KC, Lo SV, et al Trends in stroke incidence in Hong Kong differ by stroke subtype. Cerebrovasc Dis 2011; 31: 138–146. 10.1159/000321734 21135549

[15] Liu ZM, Ho SC, Tang N, Chan R, Chen YM, Woo J. Urinary sodium excretion and dietary sources of sodium intake in chinese postmenopausal women with prehypertension. PLoS One 2014; 9: e104018 10.1371/journal.pone.0104018 25083775 PMC4119001

[16] Woo J, Leung SS, Ho SC, Lam TH, Janus ED. Dietary intake and practices in the Hong Kong Chinese population. J Epidemiol Community Health 1998; 52: 631–637. 10023462 10.1136/jech.52.10.631 PMC1756630

[17] Pfeiffer E. A short portable mental status questionnaire for the assessment of organic brain deficit in elderly patients. J Am Geriatr Soc 1975; 23: 433–441. 1159263 10.1111/j.1532-5415.1975.tb00927.x

[18] Ngan MHR, Leung EMF, Kwan A, Yeung D, Chong A. A study of long term care needs patterns and impact of the elderly in Hong Kong. Research Report. 1996; Hong Kong: City University of Hong Kong.

[19] Nunnally JC. Psychometric theory (2nd Ed.). 1978; New York: McGraw-Hill.

[20] Jay M, Adams J, Herring SJ, Gillespie C, Ark T, Feldman H, et al A randomized trial of a brief multimedia intervention to improve comprehension of food labels. Prev Med 2009; 48: 25–31. 10.1016/j.ypmed.2008.10.011 19022282

[21] Centers for Disease Control and Prevention. Vital signs: food categories contributing the most to sodium consumption—United States, 2007–2008. MMWR Morb Mortal Wkly Rep 2012; 61: 92–98. 22318472

[22] Polit DF, Beck CY. The Content Validity Index: are you sure you know what’s being reported? Critique and recommendations. Res Nurs Health, 2006, 29: 489–497. 16977646 10.1002/nur.20147

[23] Leung AY, Cheung MK, Lou VW, Chan FH, Ho CK, Do TL, et al Development and validation of the Chinese Health Literacy Scale for Chronic Care. J Health Commun 2013; 18 Suppl 1: 205–222. 10.1080/10810730.2013.829138 24093357 PMC3815113

[24] Hu L, Bentler P. Cut off criteria for fit indexes in coovariance structure analysis: Coventional criteria versus new alternatives. Struct Equ Modeling 1999; 6, 1–55.

[25] Cohen J. Statistical Power Analysis for the Behavioral Sciences (2nd ed.). 1988; New Jersey: Lawrence Erlbaum.

[26] Tavakol M, Dennick R. Making sense of Cronbach’s alpha. Int J Med Educ. 2011; 2: 53–55. 28029643 10.5116/ijme.4dfb.8dfd PMC4205511

[27] Ji C, Sykes L, Paul C, Dary O, Legetic B, Campbell NR, et al Systematic review of studies comparing 24-hour and spot urine collections for estimating population salt intake. Pan American Journal of Public Health 2012; 32: 307–315. 23299293 10.1590/s1020-49892012001000010

[28] Freedman DA, Bess KD, Tucker HA, Boyd DL, Tuchman AM, Wallston KA. Public health literacy defined. Am J Prev Med 2009; 36: 446–451. 10.1016/j.amepre.2009.02.001 19362698

[29] He FJ, Li J, Macgregor GA. Effect of longer term modest salt reduction on blood pressure: Cochrane systematic review and meta-analysis of randomised trials. BMJ 2013; 346: f1325 10.1136/bmj.f1325 23558162

[30] Okuda N, Stamler J, Brown IJ, Ueshima H, Miura K, Okayama A, et al Individual efforts to reduce salt intake in China, Japan, UK, USA: what did people achieve? The INTERMAP Population Study. J Hypertens 2014; 32: 2385–2392. 10.1097/HJH.0000000000000341 25188367

